# Supplementary material for: Influence of the Great Amazon Reef System and Pleistocene sea-level drops on the phylogeography of Haemulon aurolineatum (Haemulidae)
Source: PeerJ. 2025 Aug 11;13:e19415. doi: 10.7717/peerj.19415 (PMC12352425; doi:10.7717/peerj.19415)
Supplement: Supplemental Information 1 [file peerj-13-19415-s001.docx]

| **Table S1.** Sample localities and associated information. Biogeographic provinces according with Robertson and Cramer (2014) | | | | | | | | | | | | | | | |
| --- | --- | --- | --- | --- | --- | --- | --- | --- | --- | --- | --- | --- | --- | --- | --- |
| **Biogeographic province** |  | **Country** | **Locality** | **CODE** | **Coordinates** | | **n** | **CTUM number** | **Gene Bank Accession Number** | | | | | | |
|  |  |  |  |  | **Latitude (°N)** | **Longitude (°W)** |  |  |  |  |  |  |  |  |  |
|  |  |  |  |  |  |  |  |  | ***Cox1*** | **S7** | | **Rho** | | **Myh** | |
| Northern province |  | United States | North Carolina | EUC | 34.843 | -75.721 | 5 | -------- | ANGBF23481-19 | -------- | | -------- | | -------- | |
|  |  |  |  |  |  |  |  | -------- | ANGBF23482-19 | -------- | | -------- | | -------- | |
|  |  |  |  |  |  |  |  | -------- | ANGBF23483-19 | -------- | | -------- | | -------- | |
|  |  |  |  |  |  |  |  | -------- | GBMNE42114-21 | -------- | | -------- | | -------- | |
|  |  |  |  |  |  |  |  | -------- | KF929942.1 | -------- | | -------- | | -------- | |
|  |  |  | Florida | EUF | 26.785 | -80.043 | 3 | -------- | JQ842500.1 | -------- | | -------- | | -------- | |
|  |  |  |  |  |  |  |  | -------- | JQ842501.1 | -------- | | -------- | | -------- | |
|  |  |  |  |  |  |  |  | -------- | JQ842503.1 | -------- | | -------- | | -------- | |
|  |  |  | Alabama | EUA | 30.238 | -88.100 | 1 | -------- | KF461185.1 | -------- | | -------- | | -------- | |
|  |  | Mexico (Gulf of Mexico) | Veracruz | MXV | 19.098 | -95.835 | 11 | 54261 | PQ571864 | -------- | | -------- | | -------- | |
|  |  |  |  |  |  |  |  | 54309 | PQ571865 | -------- | | -------- | | -------- | |
|  |  |  |  |  |  |  |  | 54357 | PQ571866 | -------- | | -------- | | -------- | |
|  |  |  |  |  |  |  |  | 54358 | PQ571867 | -------- | | -------- | | -------- | |
|  |  |  |  |  |  |  |  | 54376 | PQ571868 | -------- | | -------- | | -------- | |
|  |  |  |  |  |  |  |  | 54377 | PQ571869 | -------- | | -------- | | -------- | |
|  |  |  |  |  |  |  |  | 54402 | PQ571870 | PQ588478 | PQ588479 | PQ588532 | PQ588533 | PQ588560 | PQ588561 |
|  |  |  |  |  |  |  |  | 54692 | PQ571871 | -------- | | -------- | | -------- | |
|  |  |  |  |  |  |  |  | 54694 | PQ571872 | -------- | | -------- | | -------- | |
|  |  |  |  |  |  |  |  | 54764 | PQ571873 | -------- | | PQ588534 | PQ588535 | -------- | |
|  |  |  |  |  |  |  |  | 54921 | PQ571874 | PQ588480 | PQ588481 | -------- | | -------- | |
|  |  |  | Campeche | MXC | 20.211 | -91.963 | 13 | 63051 | PQ571875 | PQ588482 | PQ588483 | PQ588536 | PQ588537 | -------- | |
|  |  |  |  |  |  |  |  | 63239 | PQ571876 | PQ588486 | PQ588487 | PQ588538 | PQ588539 | PQ588564 | PQ588565 |
|  |  |  |  |  |  |  |  | 63240 | PQ571877 | PQ588484 | PQ588485 | PQ588540 | PQ588541 | PQ588562 | PQ588563 |
|  |  |  |  |  |  |  |  | 63241 | PQ571878 | -------- | | -------- | | PQ588568 | PQ588569 |
|  |  |  |  |  |  |  |  | 63242 | PQ571879 | -------- | | -------- | | -------- | |
|  |  |  |  |  |  |  |  | 63243 | PQ571880 | -------- | | -------- | | -------- | |
|  |  |  |  |  |  |  |  | 63337 | PQ571881 | -------- | | -------- | | -------- | |
|  |  |  |  |  |  |  |  | 63341 | PQ571882 | PQ588498 | PQ588499 | -------- | | -------- | |
|  |  |  |  |  |  |  |  | 63342 | PQ571883 | -------- | | -------- | | -------- | |
|  |  |  |  |  |  |  |  | 63343 | PQ571884 | PQ588496 | PQ588497 | -------- | | -------- | |
|  |  |  |  |  |  |  |  | 63344 | PQ571885 | PQ588488 | PQ588489 | PQ588542 | PQ588543 | PQ588566 | PQ588567 |
|  |  |  |  |  |  |  |  | 63345 | -------- | -------- | | -------- | | PQ588570 | PQ588571 |
|  |  |  |  |  |  |  |  | 63346 | PQ571886 | -------- | | -------- | | -------- | |
|  |  |  | Yucatán | MXY | 21.525 | -87.678 | 1 | 41746 | PQ571887 | -------- | | -------- | | -------- | |

| **Table S1.1** Continuation of table S1 | | | | | | | | | | | | | |  | |
| --- | --- | --- | --- | --- | --- | --- | --- | --- | --- | --- | --- | --- | --- | --- | --- |
| Central province |  | Mexico (Caribbean) | Quintana Roo | MXQ | 20.853 | -86.867 | 21 | 17005 | PQ571888 | PQ588494 | PQ588495 | PQ588526 | PQ588527 | PQ588574 | PQ588575 |
|  |  |  |  |  |  |  |  | 27792 | PQ571889 | PQ588492 | PQ588493 | PQ588528 | PQ588529 | PQ588572 | PQ588573 |
|  |  |  |  |  |  |  |  | 30255 | PQ571890 | PQ588500 | PQ588501 | -------- | | -------- | |
|  |  |  |  |  |  |  |  | 30256 | PQ571891 | PQ588490 | PQ588491 | -------- | | -------- | |
|  |  |  |  |  |  |  |  | 31887 | PQ571892 | -------- | | -------- | | -------- | |
|  |  |  |  |  |  |  |  | 31888 | PQ571893 | -------- | | PQ588530 | PQ588531 | PQ588576 | PQ588577 |
|  |  |  |  |  |  |  |  | 31949 | PQ571894 | -------- | | -------- | | -------- | |
|  |  |  |  |  |  |  |  | 32913 | PQ571895 | -------- | | -------- | | -------- | |
|  |  |  |  |  |  |  |  | 32937 | PQ571896 | -------- | | -------- | | -------- | |
|  |  |  |  |  |  |  |  | 33531 | PQ571897 | -------- | | -------- | | -------- | |
|  |  |  |  |  |  |  |  | 33838 | PQ571898 | -------- | | -------- | | -------- | |
|  |  |  |  |  |  |  |  | 33839 | PQ571899 | -------- | | -------- | | -------- | |
|  |  |  |  |  |  |  |  | 33843 | PQ571900 | -------- | | -------- | | -------- | |
|  |  |  |  |  |  |  |  | 33848 | PQ571901 | -------- | | -------- | | -------- | |
|  |  |  |  |  |  |  |  | 33849 | PQ571902 | -------- | | -------- | | -------- | |
|  |  |  |  |  |  |  |  | 33850 | PQ571903 | -------- | | -------- | | -------- | |
|  |  |  |  |  |  |  |  | 33851 | PQ571904 | -------- | | -------- | | -------- | |
|  |  |  |  |  |  |  |  | 33945 | PQ571905 | -------- | | -------- | | -------- | |
|  |  |  |  |  |  |  |  | 33947 | PQ571906 | -------- | | -------- | | -------- | |
|  |  |  |  |  |  |  |  | 34011 | PQ571907 | -------- | | -------- | | -------- | |
|  |  |  |  |  |  |  |  | -------- | FJMI146-10 | -------- | | -------- | | -------- | |
|  |  | Belize | Riversdale | BLR | 16.658 | -88.183 | 5 | -------- | ANGBF4086-12 | -------- | | -------- | | -------- | |
|  |  |  |  |  |  |  |  | -------- | BZLWC314-06 | -------- | | -------- | | -------- | |
|  |  |  |  |  |  |  |  | -------- | BZLWC320-06 | -------- | | -------- | | -------- | |
|  |  |  |  |  |  |  |  | -------- | JQ840858.1 | -------- | | -------- | | -------- | |
|  |  |  |  |  |  |  |  | -------- | JQ840859.1 | -------- | | -------- | | -------- | |
|  |  | Costa Rica | Manzanillo | CRM | 9.634 | -82.662 | 3 | 55688 | PQ571908 | -------- | | -------- | | -------- | |
|  |  |  |  |  |  |  |  | 55690 | PQ571909 | -------- | | -------- | | -------- | |
|  |  |  |  |  |  |  |  | 55693 | PQ571910 | -------- | | -------- | | -------- | |
|  |  | Jamaica | Falmouth | JAF | 18.869 | -77.665 | 3 | -------- | MFLE020-12 | -------- | | -------- | | -------- | |
|  |  |  |  |  |  |  |  | -------- | MFLE022-12 | -------- | | -------- | | -------- | |
|  |  |  |  |  |  |  |  | -------- | MFLE112-12 | -------- | | -------- | | -------- | |
|  |  | Puerto Rico | San Juan | PRS | 18.469 | -66.110 | 1 | 60696 | PQ571911 | -------- | | -------- | | PQ588578 | PQ588579 |
|  | | | | | | | | | | | | | | | |

| **Table S1.2** Continuation of table S1 | | | | | | | | | | | | | | | |
| --- | --- | --- | --- | --- | --- | --- | --- | --- | --- | --- | --- | --- | --- | --- | --- |
| Southern province |  | Colombia | Santa Marta | COS | 11.334 | -74.246 | 14 | 68557 | PQ571912 | PQ588504 | PQ588505 | PQ588544 | PQ588545 | PQ588580 | PQ588581 |
|  |  |  |  |  |  |  |  | 68558 | PQ571913 | PQ588506 | PQ588507 | PQ588546 | PQ588547 | PQ588582 | PQ588583 |
|  |  |  |  |  |  |  |  | 68559 | PQ571914 | -------- | | PQ588548 | PQ588549 | PQ588584 | PQ588585 |
|  |  |  |  |  |  |  |  | 68560 | -------- | -------- | | -------- | | PQ588586 | PQ588587 |
|  |  |  |  |  |  |  |  | 68562 | PQ571915 | -------- | | -------- | | -------- | |
|  |  |  |  |  |  |  |  | 68563 | PQ571916 | -------- | | -------- | | -------- | |
|  |  |  |  |  |  |  |  | 68565 | PQ571917 | PQ588514 | PQ588515 | -------- | | -------- | |
|  |  |  |  |  |  |  |  | 68566 | PQ571918 | PQ588516 | PQ588517 | PQ588550 | PQ588551 | PQ588588 | PQ588589 |
|  |  |  |  |  |  |  |  | 68567 | PQ571919 | PQ588512 | PQ588513 | -------- | | PQ588590 | PQ588591 |
|  |  |  |  |  |  |  |  | 68568 | PQ571920 | PQ588508 | PQ588509 | PQ588552 | PQ588553 | PQ588592 | PQ588593 |
|  |  |  |  |  |  |  |  | 68569 | PQ571921 | PQ588502 | PQ588503 | PQ588554 | PQ588555 | PQ588594 | PQ588595 |
|  |  |  |  |  |  |  |  | 68570 | PQ571922 | PQ588510 | PQ588511 | PQ588556 | PQ588557 | -------- | |
|  |  |  |  |  |  |  |  | 68598 | -------- | -------- | | -------- | | PQ588596 | PQ588597 |
|  |  |  |  |  |  |  |  | 68606 | -------- | -------- | | PQ588558 | PQ588559 | -------- | |
|  |  | Venezuela | Nueva Esparta | VZN | 10.95 | -63.854 | 2 | 16967 | PQ571923 | PQ588518 | PQ588519 | PQ588522 | PQ588523 | PQ588600 | PQ588601 |
|  |  |  |  |  |  |  |  | 16968 | PQ571924 | PQ588520 | PQ588521 | PQ588524 | PQ588525 | PQ588598 | PQ588599 |
|  |  | | | | | | | |  |  | |  | |  | |
| Brazilian province |  | Brazil | Bahía | BZB | -13.889°S | -38.942 | 18 | -------- | BAHIA021-14 | -------- | | -------- | | -------- | |
|  |  |  |  |  |  |  |  | -------- | BAHIA022-14 | -------- | | -------- | | -------- | |
|  |  |  |  |  |  |  |  | -------- | BAHIA023-14 | -------- | | -------- | | -------- | |
|  |  |  |  |  |  |  |  | -------- | BAHIA024-14 | -------- | | -------- | | -------- | |
|  |  |  |  |  |  |  |  | -------- | BAHIA025-14 | -------- | | -------- | | -------- | |
|  |  |  |  |  |  |  |  | -------- | BAHIA026-14 | -------- | | -------- | | -------- | |
|  |  |  |  |  |  |  |  | -------- | BAHIA027-14 | -------- | | -------- | | -------- | |
|  |  |  |  |  |  |  |  | -------- | BAHIA028-14 | -------- | | -------- | | -------- | |
|  |  |  |  |  |  |  |  | -------- | BAHIA029-14 | -------- | | -------- | | -------- | |
|  |  |  |  |  |  |  |  | -------- | BAHIA030-14 | -------- | | -------- | | -------- | |
|  |  |  |  |  |  |  |  | -------- | BAHIA031-14 | -------- | | -------- | | -------- | |
|  |  |  |  |  |  |  |  | -------- | BAHIA032-14 | -------- | | -------- | | -------- | |
|  |  |  |  |  |  |  |  | -------- | BAHIA033-14 | -------- | | -------- | | -------- | |
|  |  |  |  |  |  |  |  | -------- | BAHIA034-14 | -------- | | -------- | | -------- | |
|  |  |  |  |  |  |  |  | -------- | BAHIA035-14 | -------- | | -------- | | -------- | |
|  |  |  |  |  |  |  |  | -------- | BAHIA036-14 | -------- | | -------- | | -------- | |
|  |  |  |  |  |  |  |  | -------- | BAHIA037-14 | -------- | | -------- | | -------- | |
|  |  |  |  |  |  |  |  | -------- | BAHIA038-14 | -------- | | -------- | | -------- | |
|  | | | | | | | | | | | | | | | |

| **Table S2. Primers and PCR conditions.** | | | | | | | |
| --- | --- | --- | --- | --- | --- | --- | --- |
| **Gene Fragment** | **Primer set** | **Sequence primer** | **Denaturation step** | **Annealing step** | **Extension step** | **Final Volume reaction 12.5μl** | **Final alignment size (pb)** |
| **Mitochondrial marker** | | | | | | | |
| ***coxI***  (Ivanova, Zemlak, Hanner & Hebert, 2007) | FISH2F | 5’-TGGACTCATAAAGATGGCAC-3’ | 94°C  1min | 43.5°C  1min | 72°C  1:30min | Primer: 0.2μl  dNTP’s: 0.25μl  MgCl_2_: 1.5μl  Buffer: 1.25μl  Taq:1U | 628 |
|  | FISH2R | 3’-ACTTCAGGGTGAAAGAATCAGAA-5’ |  |  |  |  |  |
| **Nuclear markers** | | | | | | | |
| ***S7***  (Chow & Hazama, 1998) | S7RPEX1F | 5’-TGGCCTCTTCCTTGGCCGTC-3’ | 94°C  1min | 55-60°C  1min | 72°C  1:30min | Primer: 0.2μl  dNTP’s: 0.25μl  MgCl_2_: 1.5μl  Buffer: 1.25μl  Taq:1U | 406 |
|  | S7RPEX3R | 3’-GCCTTCAGGTCAGAGTTCAT-5’ |  |  |  |  |  |
| ***Rho***  (Chen, Bonillo & Lecointre, 2003) | Rh193F | 5’-CNTATGAATAYCCTCAGTACTACC-3’ | 94°C  1min | 55°C  1min | 72°C  1:30min | Primer: 0.2μl  dNTP’s: 0.25μl  MgCl_2_: 1.5μl  Buffer: 1.25μl  Taq:1U | 807 |
|  | Rh1073R | 3’-CCRCAGCACARCGTGGTGATCATG-5’ |  |  |  |  |  |
| ***Myh***  (Li, Ortí, Zhang & Lu, 2007) | My6F459 | 5’-CATMTTYTCCATCTCAGATAATGC-3’ | 94°C  1min | 53°C  1min | 72°C  1:30min | Primer: 0.2μl  dNTP’s: 0.25μl  MgCl_2_: 1.5μl  Buffer: 1.25μl  Taq:1U | 693 |
|  | Mh6R1325 | 3’-ATTCTCACCACCATCCAGTTGAA-5’ |  |  |  |  |  |

| **Table S3. Time Calibration Tree sample information.** | | | | | | |
| --- | --- | --- | --- | --- | --- | --- |
| **Genus** | **Species** | | **CTUM Number** | **Accession GenBank number** | | |
|  |  |  |  | ***coxI*** | **S7** | |
| ***Haemulon*** | *H. scudderii* | Gill, 1862 | - | EU697542.1 | JQ741862.1 | |
|  | *H. atlanticus* | Carvalho, Marceniuk, Oliveira & Wosiacki, 2020 | - | JQ365369.1 | JQ741875.1 | |
|  | *H. plumierii* | Lacepède, 1801 | - | HM389730.1 | JQ741855.1 | |
|  | *H. aurolineatum Northern* | Cuvier, 1830 | 54291 | PQ571874 | PQ588480 | PQ588481 |
|  | *H. aurolineatum Central* | Cuvier, 1830 | 17005 | PQ571888 | PQ588494 | PQ588495 |
| ***Anisotremus*** | *A. interruptus* | Gill, 1862 | - | EU697525.1 | JQ741797.1 | |
|  | *A. surinamensis* | Bloch, 1791 | - | KF929601.1 | JQ741807.1 | |
|  | *A. davidsonii* | Steindachner, 1876 | - | GU440220.1 | JQ741785.1 | |
|  | *A. scapularis* | Tschudi, 1846 | - | KY572859.1 | JQ741800.1 | |
|  | *A. taeniatus* | Gill, 1861 | - | EU697527.1 | JQ741818.1 | |
|  | *A. virginicus* | Linnaeus, 1758 | - | MN869860.1 | JQ741819.1 | |
|  | *A. caesius* | Jordan & Gilbert, 1882 | - | JQ741124.1 | JQ741783.1 | |
| CTUM: Colección de tejidos of the Colección de peces de Universidad Michoacana de San Nicolás de Hidalgo | | | | | | |


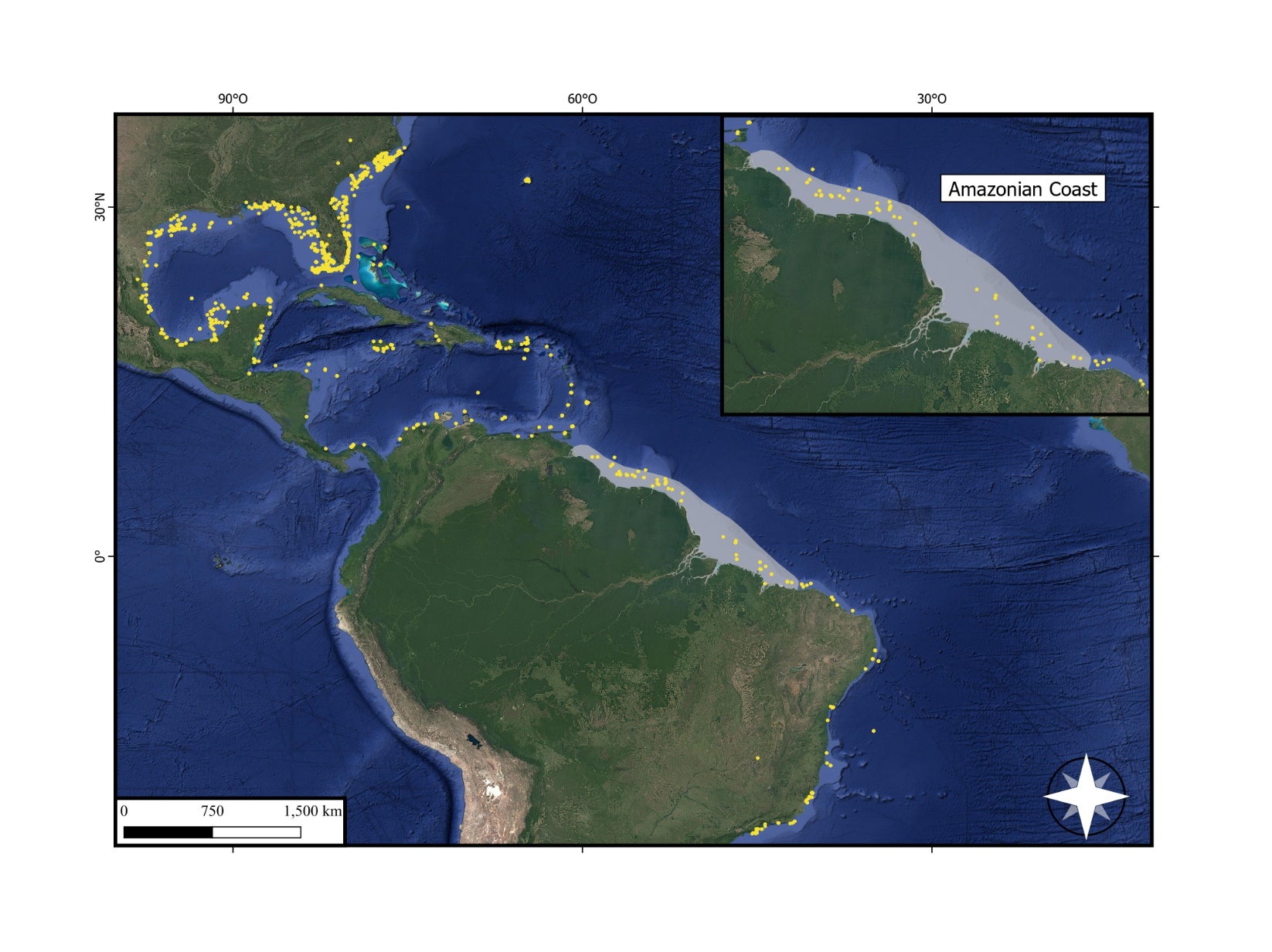


**Figure S1.** Distribution of *Haemulon aurolineatum.* Yellow points represent occurrence points of the species; grey colour denotes the delimitation of the Amazonian Reef System, retrieved and modified from Moura et al. (2016), Francini-Filho et al. (2018), Banha et al. (2022) and Carneiro et al. (2022). Satellite images for the map was retreated from © 2024 Google of QGIS.org (QGIS, 2024).

**References**

- Banha, T. N., Luiz, O. J., Asp, N. E., Pinheiro, H. T., Magris, R. A., Cordeiro, R. T., ... & Francini-Filho, R. B. (2022). The great Amazon reef system: a fact. Frontiers in Marine Science, 9, 1088956. <https://doi.org/10.3389/fmars.2022.1088956>
- Carneiro, P. B., Ximenes Neto, A. R., Jucá-Queiroz, B., Teixeira, C. E., Feitosa, C. V., Barroso, C. X., ... & Soares, M. O. (2022). Interconnected marine habitats form a single continental-scale reef system in South America. Scientific Reports, 12(1), 17359. <https://doi.org/10.1038/s41598-022-21341-x>
- Chen, W. J., Bonillo, C., & Lecointre, G. (2003). Repeatability of clades as a criterion of reliability: a case study for molecular phylogeny of Acanthomorpha (Teleostei) with larger number of taxa. Molecular phylogenetics and evolution, 26(2), 262-288.
- Chow, S., & Hazama, K. (1998). Universal PCR primers for S7 ribosomal protein gene introns in fish. Molecular ecology, 7(9), 1255-1256.
- Francini-Filho, R. B., Asp, N. E., Siegle, E., Hocevar, J., Lowyck, K., D'avila, N., ... & Thompson, F. L. (2018). Perspectives on the Great Amazon Reef: Extension, biodiversity, and threats. Frontiers in Marine Science, 5, 142. <https://doi.org/10.3389/fmars.2018.00142>
- Ivanova, N. V., Zemlak, T. S., Hanner, R. H., & Hebert, P. D. (2007). Universal primer cocktails for fish DNA barcoding. *Molecular Ecology Notes*, *7*(4), 544-548.
- Li, C., Ortí, G., Zhang, G., & Lu, G. (2007). A practical approach to phylogenomics: the phylogeny of ray-finned fish (Actinopterygii) as a case study. BMC Evolutionary Biology, 7, 1-11.
- Moura, R. L., Amado-Filho, G. M., Moraes, F. C., Brasileiro, P. S., Salomon, P. S., Mahiques, M. M., ... & Thompson, F. L. (2016). An extensive reef system at the Amazon River mouth. Science advances, 2(4), e1501252. <https://doi.org/10.1126/sciadv.1501252>
- QGIS.org (2024). QGIS Geographic Information System. QGIS Association. <http://www.qgis.org>
- Robertson, D. R., & Cramer, K. L. (2014). Defining and dividing the greater Caribbean: insights from the biogeography of shorefishes. PLoS One, 9(7), e102918. <https://doi.org/10.1371/journal.pone.0102918>
